# A Sound Source Identification Algorithm Based on Bayesian Compressive Sensing and Equivalent Source Method

**DOI:** 10.3390/s20030865

**Published:** 2020-02-06

**Authors:** Ming Zan, Zhongming Xu, Linsen Huang, Zhifei Zhang

**Affiliations:** 1 State Key Laboratory of Mechanical Transmission, Chongqing University, 174 Shazhengjie, Chongqing 400044, China; 20173201001z@cqu.edu.cn (M.Z.); 20183201008@cqu.edu.cn (L.H.); z.zhang@cqu.edu.cn (Z.Z.); 2 College of Automotive Engineering, Chongqing University, 174 Shazhengjie, Chongqing 400044, China

**Keywords:** near-field acoustical holography, equivalent source method, Bayesian compressive sensing, functional beamforming

## Abstract

Near-field acoustic holography (NAH) based on equivalent source method (ESM) is an effective method for identifying sound sources. Conventional ESM focuses on relatively low frequencies and cannot provide a satisfactory solution at high frequencies. So its improved method called wideband acoustic holography (WBH) has been proposed, which has high reconstruction accuracy at medium-to-high frequencies. However, it is less accurate for coherent sound sources at low frequencies. To improve the reconstruction accuracy of conventional ESM and WBH, a sound source identification algorithm based on Bayesian compressive sensing (BCS) and ESM is proposed. This method uses a hierarchical Laplace sparse prior probability distribution, and adaptively adjusts the regularization parameter, so that the energy is concentrated near the correct equivalent source. Referring to the function beamforming idea, the original algorithm with order *v* can improve its dynamic range, and then more accurate position information is obtained. Based on the simulation of irregular microphone array, comparisons with conventional ESM and WBH show that the proposed method is more accurate, suitable for a wider range of frequencies, and has better reconstruction performance for coherent sources. By increasing the order *v*, the coherent sources can be located accurately. Finally, the stability and reliability of the proposed method are verified by experiments.

## 1. Introduction

Acoustic holography is an advanced technology for sound source identification and sound field visualization. Williams and Maynard put forward the generalized theory of acoustic holography in 1980s [1]. The technique requires holographic data to be measured in the near-field of the sound source, so it is also called near-field acoustical holography (NAH). Since this method based on two-dimensional spatial Fourier transform is mainly applied to regular measurement surfaces, it has limitations in engineering applications.

To reduce the limitation of measurement conditions, in the past few decades various NAH methods have been developed, such as the boundary element method (BEM) [2,3], the statistically optimized near-field acoustic holography (SONAH) [4,5], and the equivalent source method (ESM) [6,7]. ESM, also known as wave superposition method (WSM), is an efficacious method for identifying sound sources. For ESM, it assumes that the sound field is a superposition of some equivalent sound sources and approximates the boundary conditions by placing several simple sound sources. The calculation error and robustness analysis results of ESM show that it is an effective method with high precision [6]. Conventional ESM usually uses $l_{2}$ norm regularization method, represented by Tikhonov regularization method (TRESM), to find the least energy solution in the solution subspace. It provides good resolution at low frequencies, but the reconstruction accuracy is low at mid-and-high frequencies. To overcome this problem, J. Hald proposed the wideband acoustic holography (WBH) method [8,9], which uses the steepest descent method combining dynamic filtering to obtain a sparse solution of equivalent source strength, with high reconstruction accuracy at mid-and-high frequencies. It has high computational efficiency and can be applied in irregular planar arrays. However, for coherent sound sources, the resolution of WBH at low frequencies is restricted due to the average spacing of array microphones.

In recent years, as an important tool for improving resolution, many sparse regularization methods have attracted the attention of scholars and been widely used in the field of image processing [10,11]. The sparse regularization method has also been applied to the field of sound source identification. Compressive sensing (CS) [12,13,14] based on sparse representation is a technique of signal acquiring and processing whose basic assumption is that data usually has sparse representations on certain bases and can be described with a few non-zero coefficients. In acoustics, it is often meaningful to obtain a sparse representation of the sound field. Chardon et al. [15] combined the sparsity and CS theory with NAH to identify the sound source with fewer microphones. Since the assumption of CS technology is the sparsity of the signal, the CS-based NAH can reconstruct high-resolution sound sources with less measured values. N. Chu et al. [16] combined aeroacoustic imaging with Bayesian compressive sensing (BCS) and made a comparison with the conventional beamforming method, such as DAMAS, CLEAN, etc., showing that the Bayesian method has a wider dynamic display range and it is more robust. In addition, there are some other methods [17,18,19,20,21] to prove the importance of regularization and CS framework.

The purpose of this paper is to further improve the reconstruction accuracy of WBH and conventional ESM methods, and an alternative ESM-based algorithm is proposed to reconstruct sound field information more accurately. Compared with the conventional ESM model, a near-field acoustic holography model based on ESM and BCS is established firstly. Then the independent Laplace prior is utilized for each equivalent source strength in a hierarchical manner. In the process of finding optimal sources, an effective greedy construction algorithm [14] is used to continuously update the regularization parameter in the iteration to broaden the equivalent source dynamic range. Since the parameters required in the model are completely estimated automatically during the calculation, the algorithm is fully automated. Finally, the equivalent source strength is estimated by the distribution of the equivalent source strength. For the sake of brevity, this BCS-based method using Laplace priors is referred to as LPBCS in the following article. In addition, referring to the function beamforming idea [22,23], the original algorithm is extended to the high order *v* form (LPBCS-*v* for brevity), and its dynamic display range is significantly improved, thereby obtaining more accurate information of positioning.

The outline of this paper is as follows. Firstly, the application of NAH and CS in the field of sound source identification are simply introduced. Then, Section 2 presents the basic theory of ESM, TRESM, and WBH. Section 3 presents the theoretical description of LPBCS and its extension form LPBCS-*v*. In Section 4, numerical simulations are performed to evaluate the proposed method by comparing it with the TRESM and WBH. Section 5 further validates the effectiveness of the proposed method through experiments. Finally, in Section 6 the conclusions are summarized.

## 2. Theoretical Background

### 2.1. Equivalent Source Model

**Figure 1:**
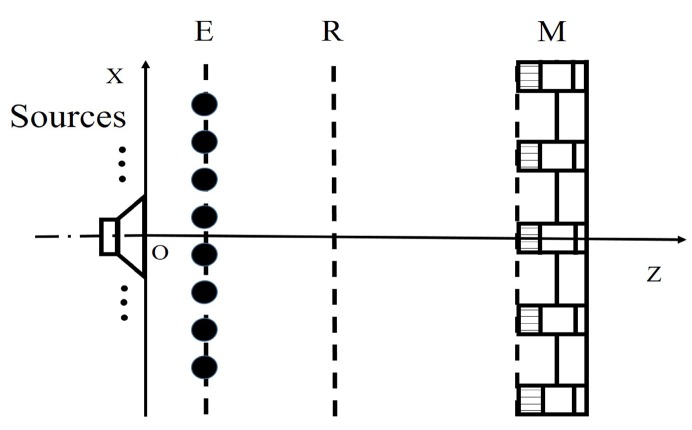
Equivalent source diagram with equivalent source surface E, reconstruction surface R, and measurement surface M.

The basic idea of the NAH based on ESM is that the sound field radiated by actual sound sources can be modeled by the superposition of a series of simple monopoles. The spatial sound field can be reconstructed by solving the strength of each equivalent source, as shown in Figure 1. The equivalent source surface is constructed near the actual sound sources. It assumes that the equivalent source radiates the sound pressure outward by the free-field radiation law. The sound pressure measured by the microphone array is used as the boundary condition to establish the equation. The equivalent source strength can be obtained by solving the inverse problem equation so that it is further possible to reconstruct the sound field information on the reconstruction surface. To simplify the calculation, the equivalent source and reconstruction surfaces are set to be rectangular planes. Assuming that there are *H* microphones on the measurement surface and *L* virtual equivalent sources on the equivalent source surface, the theoretical calculation model can be obtained

(1) $$\left[\begin{array}{c} p_{1}\\ p_{2}\\ \vdots \\ p_{H} \end{array}\right]=\left[\begin{array}{ccccccc} G_{11} & \;\; & G_{12} & \;\; & \cdots & \;\; & G_{1L}\\ G_{21} & \;\; & G_{22} & \;\; & \cdots & \;\; & G_{2L}\\ \vdots & \;\; & \vdots & \;\; & \ddots & \;\; & \vdots \\ G_{H1} & \;\; & G_{H2} & \;\; & \cdots & \;\; & G_{HL} \end{array}\right]\left[\begin{array}{c} q_{1}\\ q_{2}\\ \vdots \\ q_{L} \end{array}\right].$$

Complex numbers $q_{l}$ and $p_{h}$ are the strength of the equivalent source and measured pressure, respectively. $G_{hl}$ can describe the connection between $q_{l}$ and $p_{h}$. It can be expressed as

(2) $$G_{hl}=\frac{\exp(-jk{∥r_{h}-r_{l}∥}_{2})}{4\pi {∥r_{h}-r_{l}∥}_{2}},$$

where *k* is the wave number, $r_{h}$ and $r_{l}$ are the Cartesian coordinate of the *h*-th microphone and the *l*-th equivalent source, respectively. ${∥r_{h}-r_{l}∥}_{2}$ is the Euclidean distance of the vector difference. Equation (1) can be simplified as matrix-vector form

(3) p=Gq.

Here, G is called the Green matrix. When the calculation of equivalent source strength is completed, the reconstruction surface sound pressure can be expressed as

(4) $$\tilde{p}=\tilde{G}q.$$

Similar to the Green matrix G, $\tilde{G}$ represents the transfer matrix between the equivalent source surface grids and the reconstruction surface grids. $\tilde{p}$ indicates the reconstruction surface sound pressure. It can be seen that the accuracy of the calculated strength of equivalent sources will greatly affect the accuracy of the reconstructed sound pressure of the reconstruction surface, so the method used to calculate the equivalent source strength is crucial.

However, in general engineering applications, the number of measurement points is often much less than the number of virtual equivalent sources due to the limitation of measurement conditions. The problem is severely ill-conditioned and usually underdetermined and has a non-unique solution subspace. It is necessary to constrain the equivalent source strength vector so that it has a meaningful solution.

### 2.2. Tikhonov Regularization and Wideband Acoustical Holography

In general, limiting the solution vector q requires regularization methods. The Tikhonov regularization method is one of the effective methods for calculating the optimal solution of the equivalent source strength. TRESM usually adds an $l_{2}$ norm penalty to the solution vector q when minimizing the residual norm ${∥p-Gq∥}_{2}^{2}$. Since the result given by the Tikhonov regularization method is only a least squares solution, some ghost sources appear near the real sound source. Therefore, it causes the sound source image to become blurred at the mid-and-high frequencies. To suppress the number of ghost sources, WBH uses the steepest descent iterative algorithm to solve the problem. In each iteration, the threshold filtering process is used to set elements whose values are smaller than the threshold value to zero, while retaining elements above the threshold value. At the same time, the threshold value decreases as the number of iterations increases, thereby reducing its own effect. The threshold filtering process can effectively reduce the number of equivalent sources, so that the energy is more concentrated near the true equivalent source. However, WBH can accurately identify the coherent sound sources only when the microphone spacing in the array is appropriate. It is recommended to identify the coherent sound sources above 0.7 times the frequency of a half wavelength average array microphones spacing.

Therefore, in this paper, we focus on developing a method to solve the ESM-based sound sources identification problem in a Bayesian framework, which can better identify the sound source. This method enables the coherent sound sources to be accurately identified at relatively low frequencies while maintaining high frequencies accuracy. In addition, taking advantage of the function beamforming properties, for the problem with low resolution of coherent sound sources, the cross-spectral matrix is used to greatly increase the dynamic display range by increasing the order, which makes the sound source location more accurate.

## 3. Reconstruction Algorithm

### 3.1. Compressive Sensing Model for Sound Field Reconstruction

In the compressive sensing problem, it assumes that for a signal $d\in R^{N}$, it can be represented by a set of orthogonal basis $d=\sum_{i}\varphi_{i}x_{i}=\Phi x$, where Φ is the basis matrix and $x\in R^{N}$ is a sparse vector, i.e., most of its coefficients are zero. Then the *M*-dimensional observation vector $y\in R^{M}$ is obtained by the projection matrix Ψ, i.e.y=Ψd=ΨΦx=Ax. When the measurements are noisy, the form is as follows

(5) y=Ax+n.

Since x is compressible, its reconstruction can be defined as a sparse regularization problem as follows

(6) $$\hat{x}=\arg\min_{x}\left\{{∥y-Ax∥}_{2}^{2}+\rho {∥x∥}_{1}\right\},$$

where the term ρ called regularization parameter is significant and it controls the relative importance applied to the Euclidian error and the sparsity of x.

In Bayesian framework, the sparse vector x can be estimated from the CS-based measured value y. The posterior probability distribution function of the parameters is first calculated, and then the statistics in the distribution are used to estimate the specific required values.

In order to apply the Bayesian compressive sensing method to sound field reconstruction, the strategy is to convert the complex vector and matrix into real values, that is, to decompose the complex values into real and imaginary parts. Both parts have the same sparsity. Then the sound field reconstruction problem is the same as Equation (5) with

(7) y=Re(p)Im(p),

(8) x=Re(q)Im(q),

(9) A=Re(G)−Im(G)Im(G)Re(G).

### 3.2. Reconstruction via Bayesian Method

In the reconstruction process, the observation model and the signal model are first introduced, and then sparse Bayesian inference is performed which includes a fast greedy algorithm.

In this the observation model, it assumes that the observed noise is independent and a Gaussian distribution with zero mean and the variance $s_{0}$. Therefore, the probability distribution of the measured vector y is

(10) $$P\left(y|x,s_{0}^{-1}\right)=N\left(y|Ax,s_{0}\right),$$

where $s_{0}^{-1}$ is the inverse-variance or precision of observed noise. Because the conjugate probability distribution of Gaussian distribution precision is Gamma distribution, there is

(11) $$P\left(s_{0}^{-1}|\alpha ,\beta \right)=\Gamma \left(s_{0}^{-1}|\alpha ,\beta \right).$$

The conjugate prior makes the functional form of the posterior probability the same as the prior probability, which greatly simplifies the Bayesian analysis.

Then the signal model is introduced. Due to the sparsity of the equivalent sources, the unknown parameter x is a sparse vector. According to the Bayesian method, a hierarchical prior is introduced due to the sparse prior probability of x. As the first layer of the hierarchical model, the Gaussian distribution is employed on x

(12) $$P\left(x|s\right)=\prod_{i=1}^{N}N\left(x_{i}|0,s_{i}\right).$$

As a second layer of the hierarchical model, the following Gamma distribution is used on the hyper-parameter $s_{i}$,

(13) $$P\left(s_{i}|\lambda \right)=\Gamma \left(s_{i}|1,\frac{\lambda }{2}\right)=\frac{\lambda }{2}\exp\left(\frac{-\lambda s_{i}}{2}\right).$$

The overall prior on x is evaluated as

(14) $$P\left(x|\lambda \right)=\int P\left(x|s\right)P\left(s|\lambda \right)ds=\frac{\lambda^{N/2}}{2^{N}}\exp\left(-\sqrt{\lambda }\sum_{i=1}^{N}|x_{i}|\right).$$

In the last layer, as a hyper-parameter, λ has the following hyper-prior distribution

(15) $$P\left(\lambda |\theta \right)=\Gamma \left(\lambda |\frac{\theta }{2},\frac{\theta }{2}\right).$$

The parameter selection of the prior distribution of λ is very flexible. When θ approaches to zero, the distribution provides a very ambiguous message. The hierarchical prior described above has a multi-layer combination. The first two layers constitute the Laplace prior distribution, which has many advanced properties, such as heavy tails, the sharp peak at zero and the shape regulated by parameters. And the last layer can be used to calculate λ.

Here, Bayesian inference is performed by using type-II maximum likelihood approach. In the process of reconstruction, it is the posterior distributions of parameters that need to be solved. According to Bayesian inference, there is the posterior distribution

(16) $$P\left(x,s,\lambda ,s_{0}^{-1}|y\right)=\frac{P(x,s,\lambda ,s_{0}^{-1},y)}{P(y)}.$$

Here, the dependency on θ is dropped for conciseness. Due to the hierarchical prior structure, the joint distribution can be defined as

(17) $$P\left(x,s,\lambda ,s_{0}^{-1},y\right)=P\left(y|x,s_{0}^{-1}\right)P\left(x|s\right)P\left(s|\lambda \right)P\left(\lambda \right)P\left(s_{0}^{-1}\right).$$

The denominator of the posterior formulation is not easy to solve because it requires complicated integration and has no analytical solution. So Equation (16) needs to be properly decomposed. The decomposition used in this inference procedure is as follows

(18) $$P\left(x,s,\lambda ,s_{0}^{-1}|y\right)=P\left(x|y,s,\lambda ,s_{0}^{-1}\right)P\left(s,\lambda ,s_{0}^{-1}|y\right),$$

$P(x|y,s,\lambda ,s_{0}^{-1})$ is the posterior probability distribution on x which can be expressed as a Gaussian distribution N(x|μ,Σ) with mean and variance

(19) $$\begin{array}{c} \mu =\Sigma s_{0}^{-1}A^{T}y, \end{array}$$

(20) $$\begin{array}{c} \Sigma ={\left(S^{-1}+s_{0}^{-1}A^{T}A\right)}^{-1}, \end{array}$$

where $S=diag(s_{1},s_{2},\cdots ,s_{N})$. Since $P\left(s,\lambda ,s_{0}^{-1}|y\right)\propto P\left(y,s,\lambda ,s_{0}^{-1}\right)$, the hyper-parameters s, λ, $s_{0}^{-1}$ can be estimated by maximizing the joint distribution $P(y,s,\lambda ,s_{0}^{-1})$, which can be obtained from Equation (17) by integrating out x

(21) $$P\left(y,s,\lambda ,s_{0}^{-1}\right)={\left(2\pi \right)}^{-\frac{N}{2}}{\left|C\right|}^{-\frac{1}{2}}\exp\left(\frac{-y^{T}C^{-1}y}{2}\right)P\left(s|\lambda \right)P\left(\lambda \right)P\left(s_{0}^{-1}\right),$$

where $C=s_{0}I+{ASA}^{T}$. To facilitate calculations, maximize the logarithm of Equation (21) equivalently

(22) $$\begin{array}{cc} L= & -\frac{N}{2}\log\left(2\pi \right)-\frac{\log|C|}{2}-\frac{y^{T}C^{-1}y}{2}+N\log\frac{\lambda }{2}-\sum_{i=1}^{N}\frac{\lambda s_{i}}{2}+\frac{\theta }{2}\log\frac{\theta }{2}\\ \; & -\log\Gamma \left(\frac{\theta }{2}\right)+\left(\frac{\theta }{2}-1\right)\log\lambda -\frac{\theta \lambda }{2}-\left(\alpha -1\right)\log s_{0}-\frac{\beta }{s_{0}}. \end{array}$$

To reduce the computational complexity, a greedy construction manner can be used to decompose the C into

(23) $$C=s_{0}I+ASA^{T}=s_{0}I+\sum_{j\neq i}s_{j}a_{j}a_{j}^{T}+s_{i}a_{i}a_{i}^{T}=C_{-i}+s_{i}a_{i}a_{i}^{T}.$$

where $a_{i}$ is the *i*-th column basis in A, and $C_{-i}$ is C with $a_{i}$ removed. By using wood-bury identity and determinant identity, $C^{-1}$ and |C| can be obtained

(24) $$\begin{array}{c} C^{-1}=C_{-i}^{-1}-\frac{C_{-i}^{-1}a_{i}a_{i}^{T}C_{-i}^{-1}}{s_{i}^{-1}+a_{i}^{T}C_{-i}^{-1}a_{i}}, \end{array}$$

(25) $$\begin{array}{c} \left|C|=\right|C_{-i}||1+s_{i}a_{i}^{T}C_{-i}^{-1}a_{i}|. \end{array}$$

Then L can be treated as a function of s only

(26) $$\begin{array}{cc} L(s) & =-\frac{1}{2}\left(N\log\left(2\pi \right)+\log\left|C_{-i}\right|+y^{T}C_{-i}^{-1}y+\sum_{j\neq i}\frac{\lambda s_{j}}{2}\right)+\frac{1}{2}\left(\log\frac{1}{1+s_{i}f_{i}}+\frac{g_{i}^{2}s_{i}}{1+s_{i}f_{i}}-\lambda s_{i}\right)\\ \; & =L\left(s_{-i}\right)+l\left(s_{i}\right). \end{array}$$

where $f_{i}$ and $g_{i}$ are defined as

(27) $$\begin{array}{c} f_{i}=a_{i}^{T}C_{-i}^{-1}a_{i}, \end{array}$$

(28) $$\begin{array}{c} g_{i}=a_{i}^{T}C_{-i}^{-1}y. \end{array}$$

The term $l(s_{i})$ containing parameter $s_{i}$ in Equation (26) is separated from others so that the derivation with respect to $s_{i}$ is simplified. By setting the derivation to zero, its solution can be expressed analytically as

(29) $$s_{i}=\left\{\begin{array}{cc} \frac{-f_{i}\left(f_{i}+2\lambda \right)+f_{i}\sqrt{{\left(f_{i}+2\lambda \right)}^{2}-4\lambda \left(f_{i}-g_{i}^{2}+\lambda \right))}}{2\lambda f_{i}^{2}}, & \mathrm{if}\;g_{i}^{2}-f_{i}>\lambda \\ 0, & \mathrm{if}\;g_{i}^{2}-f_{i}\leq \lambda . \end{array}\right.$$

The updates of other hyper-parameters are similar. By taking the derivative of Equation (26) with respect to λ and making it equal to zero, the update formula of the parameter λ can be obtained

(30) $$\lambda =\frac{2N-2+\theta }{\sum_{i}s_{i}+\theta }.$$

Similarly, an estimate of the noise variance can be written as

(31) $$s_{0}=\frac{{∥y-A\mu ∥}_{2}^{2}+2\beta }{N+2\alpha }.$$

The regularization parameter ρ in Equation (6) is driven by the parameters λ and $s_{0}$ [13], so the iteration process of λ and $s_{0}$ is equivalent to iteratively updating the regularization parameter ρ which can make the reconstruction of sound field more accurate. This is an important reason to introduce a third layer of the hierarchical prior to overcome the drawback of using a fixed regularization parameter in conventional ESM.

So far, through the above Bayesian inference, the updated formulas of the necessary hyper-parameters have been obtained. The parameter μ is the mean of the posterior distribution of x. It is used to estimate the vector x. Then the equivalent source strength q can be obtained from Equation (8). The LPBCS algorithm is summarized as follows:

1. Input y and A by Equation (7) and Equation (9).
2. Initialize $s_{0}=0.1\mathrm{var}\left(y\right)$, s=0, λ=0.
3. Select a basis vector $a_{i}$ out of A and update the basis vector,
  if $g_{i}^{2}-f_{i}>\lambda$ and $s_{i}>0$, $a_{i}$ is in the model, re-estimate $s_{i}$,
  if $g_{i}^{2}-f_{i}>\lambda$ and $s_{i}=0$, add $a_{i}$ to the model with updated $s_{i}$,
  if $g_{i}^{2}-f_{i}\leq \lambda$, prune $a_{i}$ from the model and set $s_{i}=0$.
4. Update parameters μ, Σ, $g_{i}$, $f_{i}$, λ.
5. The iteration terminates when the value of marginal likelihood changes less than the set threshold or when the maximum number of iterations is exceeded. Otherwise go to step 3.
6. Set x=μ, then estimate the vector q by Equation (8).

### 3.3. High Dynamic Range for LPBCS-*v*

To locate the sound source more accurately, the dynamic range is improved. Inspired by the function beamforming, the solution of Equation (4) is modified, and the output at the field point t on the reconstruction surface can be represented by the cross-spectral matrix of q,

(32) $${\tilde{P}}_{t}={\tilde{p}}_{t}{\tilde{p}}_{t}^{H}={\tilde{G}}_{t}qq^{H}{\tilde{G}}_{t}^{H}={\tilde{G}}_{t}C{\tilde{G}}_{t}^{H},$$

where ${\tilde{G}}_{t}$ is the *t*-th row vector of $\tilde{G}$, $C=qq^{H}$. The eigendecomposition of C can be written as

(33) $$C=U\Lambda U^{H},$$

where U is a unitary matrix which represents the set of eigenvectors of C, Λ is a diagonal matrix $diag(\gamma_{1},\gamma_{2},\cdots ,\gamma_{N})$ whose diagonal elements show the eigenvalues. An exponential matrix function of C is defined by

(34) $$C^{1/v}=Udiag(\gamma_{1}^{1/v},\gamma_{2}^{1/v},\cdots ,\gamma_{N}^{1/v})U^{H},$$

where 1/v is the power of the eigenvalue. Then replace C in Equation (32) with $C^{1/v}$ and take the *v* power of the output

(35) $${\tilde{P}}_{t}^{\prime }={\left({\tilde{G}}_{t}C^{1/v}{\tilde{G}}_{t}^{H}\right)}^{v}.$$

Finally, the output can be written in vector form ${\tilde{P}}_{v}={\left[{{\tilde{P}}^{\prime }}_{1},\cdots ,{{\tilde{P}}^{\prime }}_{t},\cdots ,{{\tilde{P}}^{\prime }}_{N}\right]}^{T}$. By applying the order *v*, the output strength of the sound source may be smaller than the true value, so the amplitude is corrected to ensure the true estimation of the maximum pressure amplitude

(36) $${\tilde{P}}_{out}={\tilde{P}}_{v}\frac{\max(|\tilde{p}|)}{\max(|{\tilde{P}}_{v}|)}.$$

This is the final output of LPBCS-*v*. The order v=0 is defined as the original output. If we want to ensure a near-perfect reconstruction of the sound field, we can apply the result when the order is zero. However, if we want to identify the sound source position with high precision and large dynamic range, the order is adjusted to 1, 2, 3, etc. Therefore, the selection of the order *v* is very flexible and can provide high-precision sound source localization.

## 4. Numerical Simulations

**Figure 2:**
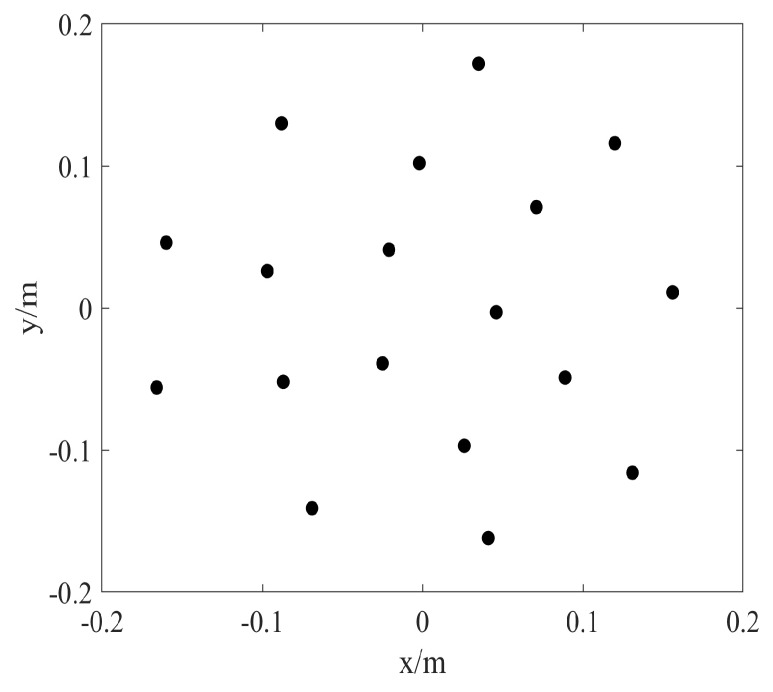
The 18-channel B&K Combo microphone array [24,25].

To illustrate the performance of the proposed method for sound source identification, numerical simulations are implemented. The specific settings of the simulation are as follows. In the same coordinate system, the distance between the measurement surface and the origin of the coordinate is 0.2 m, and the distances from the equivalent source surface and the reconstruction surface to the origin are 0.001 m and 0.02 m, respectively. These surfaces are parallel to each other and consistent with Figure 1. Assume that each sound source is a pulsating sphere with a radius of 0.01 m and vibrating velocity of $2.5\times 10^{-2}$ m/s. The speed of sound is 340 m/s. Reconstruction surface and the equivalent source surface are both meshed into 41×41 points with the grid spacing of 0.02 m. To ensure consistency between simulations and experimentations, a Combo microphone array is used for simulation measurements. The array has 18 microphones distributed in a circle, and the microphone coordinates of the measurement array are shown in Figure 2.

**Figure 3:**
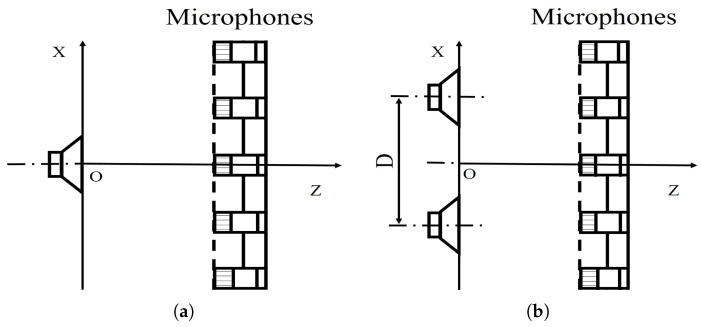
The setup of the simulations for the source reconstruction. (**a**) One single source; (**b**) Coherent sources.

**Figure 4:**
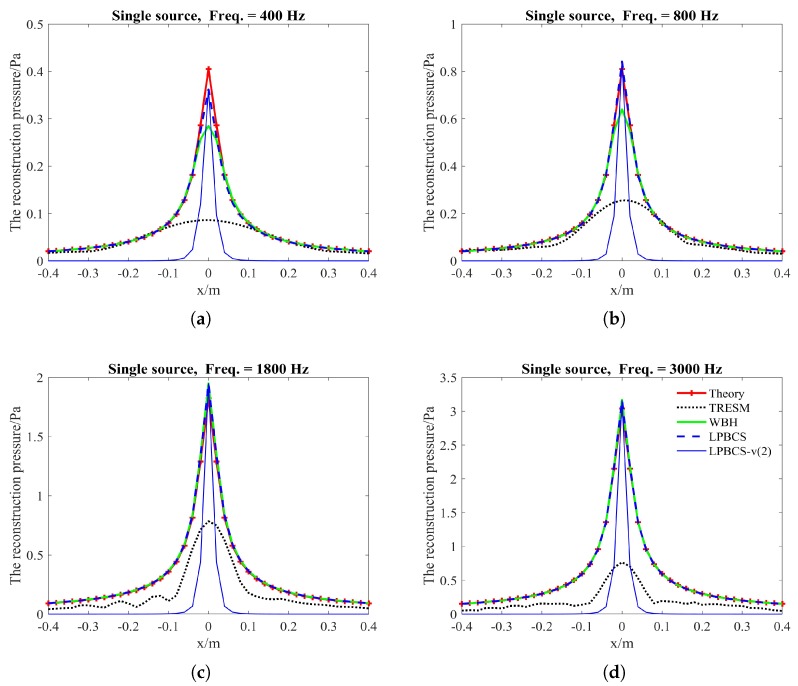
The reconstructed pressure of single source along the middle row of reconstruction surface at different frequencies. SNR = 20 dB. (**a**) 400 Hz; (**b**) 800 Hz; (**c**) 1800 Hz; (**d**) 3000 Hz.

**Figure 5:**
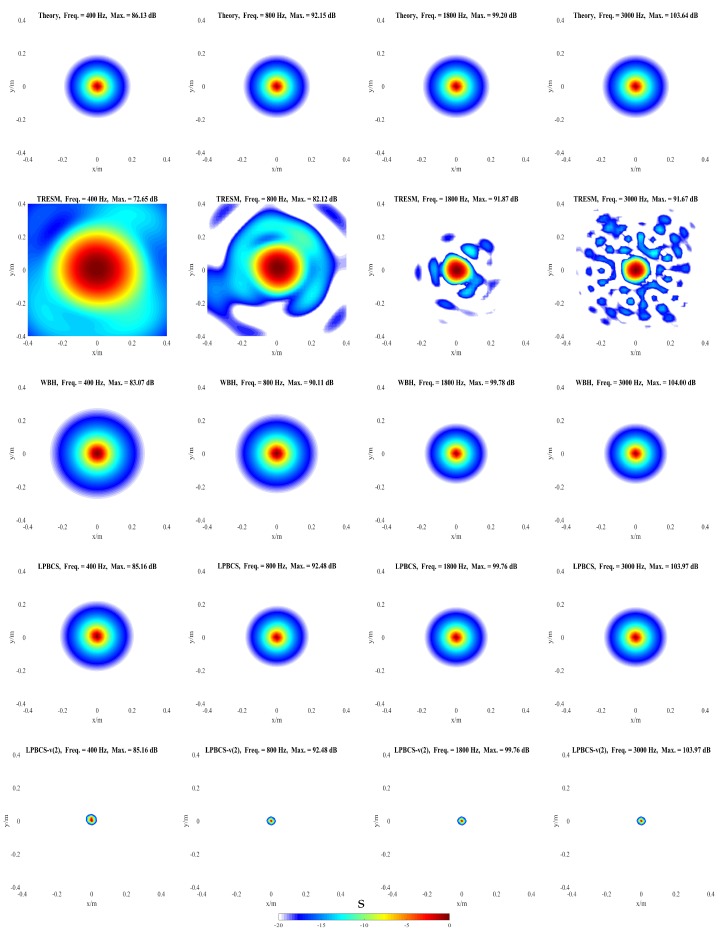
Simulated sound source maps for single source at 400 Hz (**the first column**), 800 Hz (**the second column**), 1800 Hz (**the third column**), 3000 Hz (**the fourth column**). For each column, from top to bottom are the Theory, TRESM, WBH, LPBCS, and LPBCS-v(2). SNR = 20 dB.

**Figure 6:**
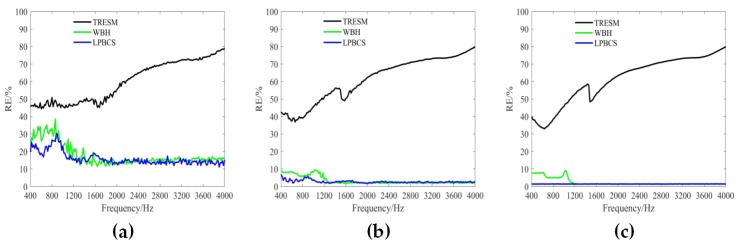
The curves of single source reconstruction error under different measurement noise levels. The source is 0.2 m from the array. (**a**) SNR = 10 dB; (**b**) SNR = 20 dB; (**c**) SNR = 30 dB.

**Figure 7:**
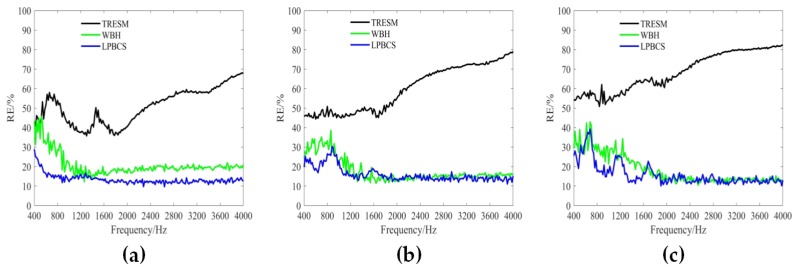
The curves of single source reconstruction error at different measurement distances. SNR = 10 dB. (**a**) 0.1 m; (**b**) 0.2 m; (**c**) 0.3 m.

**Figure 8:**
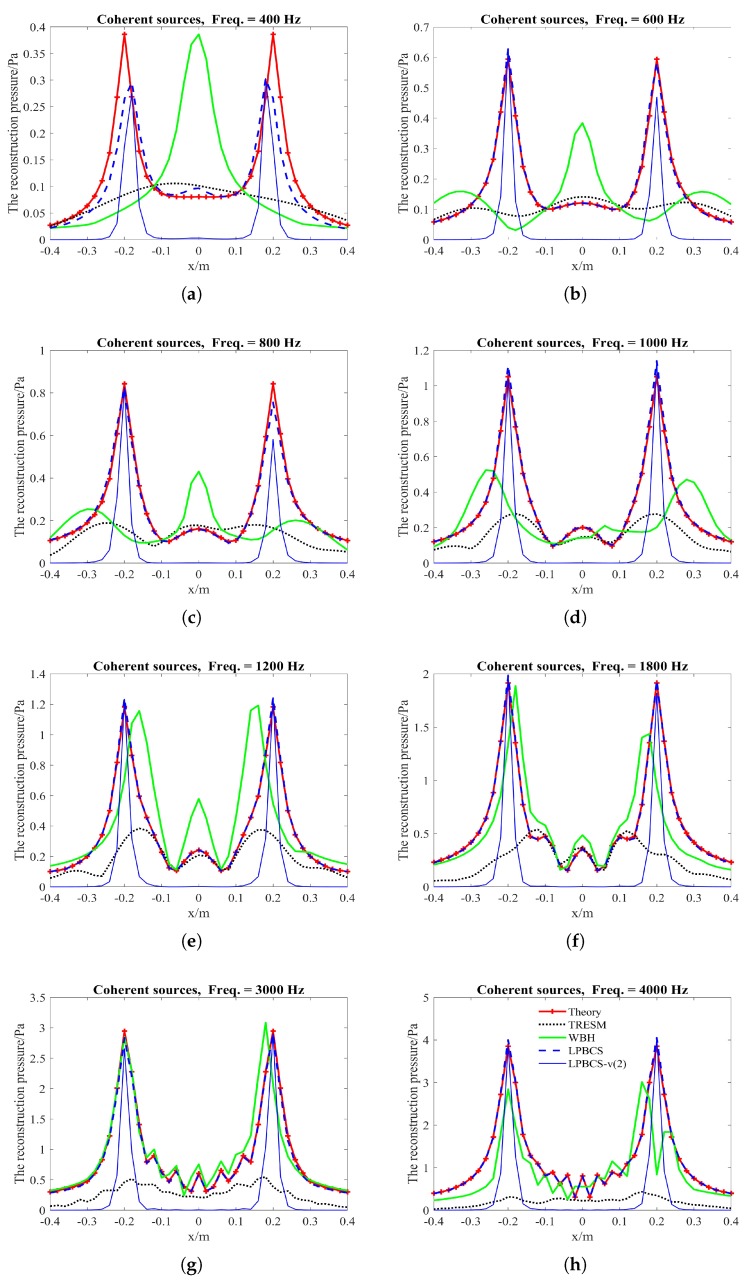
The reconstructed pressure of coherent sources along the middle row of reconstruction surface at different frequencies. SNR = 20 dB. (**a**) 400 Hz; (**b**) 600 Hz; (**c**) 800 Hz; (**d**) 1000 Hz; (**e**) 1200 Hz; (**f**) 1800 Hz; (**g**) 3000 Hz; (**h**) 4000 Hz.

**Figure 9:**
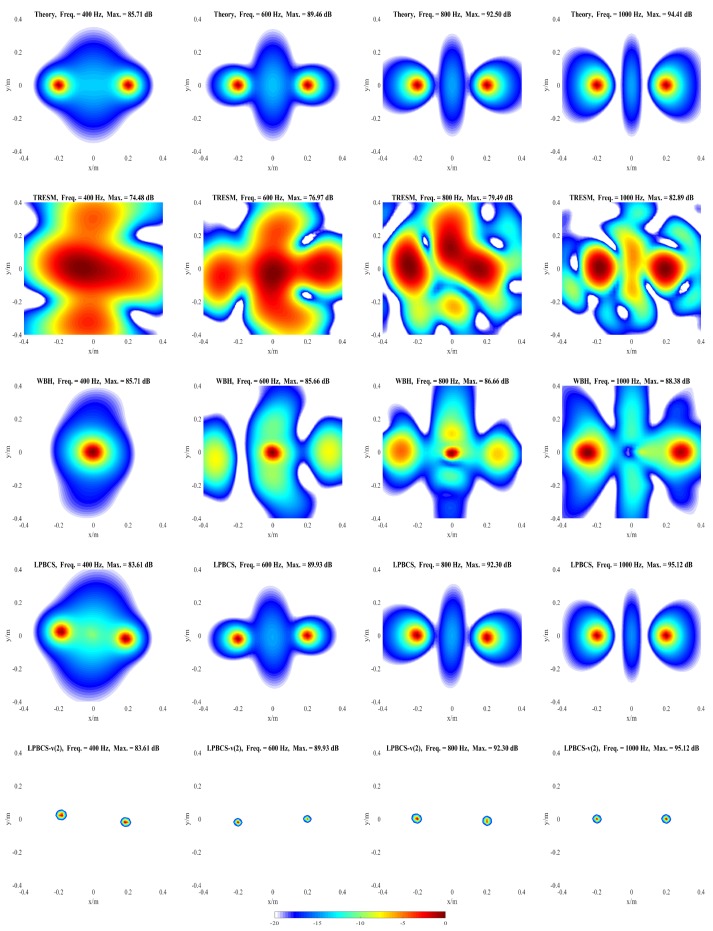
Simulated sound source maps for coherent sources at 400 Hz (**the first column**), 600 Hz (**the second column**), 800 Hz (**the third column**), 1000 Hz (**the fourth column**). For each column, from top to bottom are the Theory, TRESM, WBH, LPBCS, and LPBCS-v(2). SNR = 20 dB.

**Figure 10:**
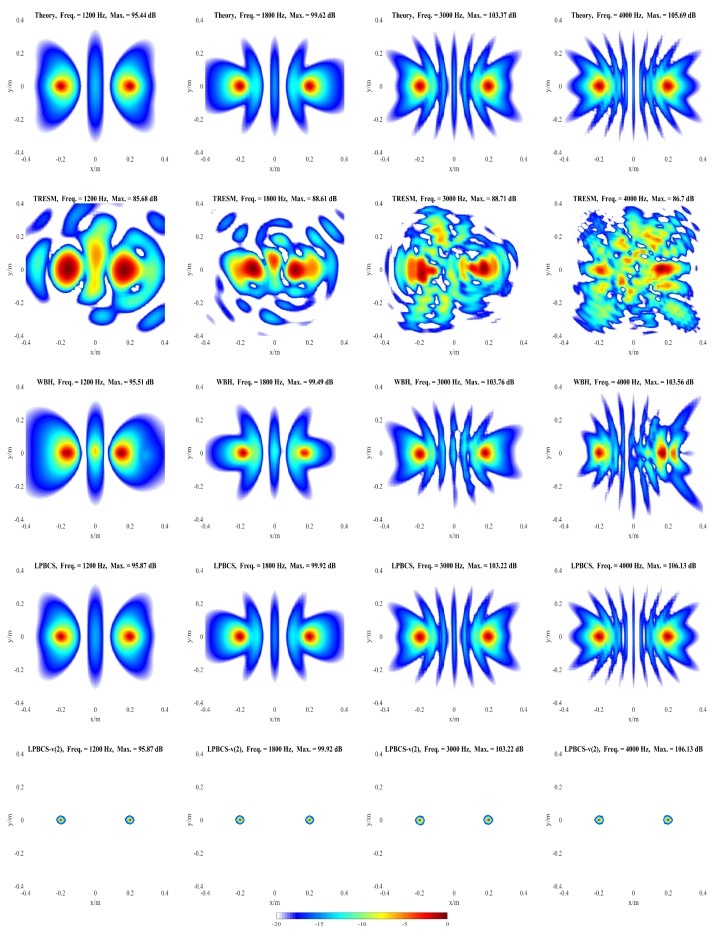
Simulated sound source maps for coherent sources at 1200 Hz (**the first column**), 1800 Hz (**the second column**), 3000 Hz (**the third column**), 4000 Hz (**the fourth column**). For each column, from top to bottom are the Theory, TRESM, WBH, LPBCS, and LPBCS-v(2). SNR = 20 dB.

**Figure 11:**
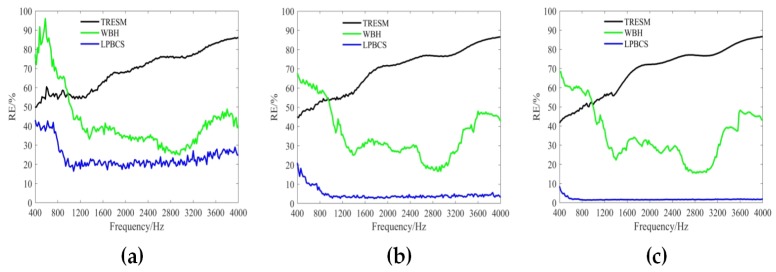
The curves of coherent sources reconstruction error under different measurement noise levels. Sources are 0.2 m from the array. (**a**) SNR = 10 dB; (**b**) SNR = 20 dB; (**c**) SNR = 30 dB.

**Figure 12:**
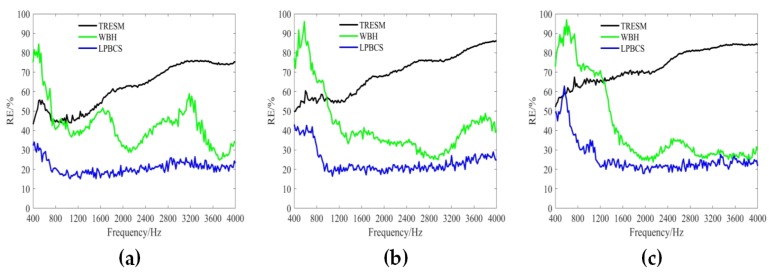
The curves of coherent sources reconstruction error at different measurement distances. SNR = 10 dB. (**a**) 0.1 m; (**b**) 0.2 m; (**c**) 0.3 m.

The influences of different factors on the reconstruction accuracy are studied under two simulation conditions. In condition one, a pulsating sphere is placed at (0, 0, 0) m, which is the middle of the sound source surface as shown in Figure 3a, and the simulation results are presented in Figure 4, Figure 5, Figure 6 and Figure 7. In condition two, to study the identification of coherent sound sources, two coherent pulsating spheres are placed at coordinates (0.2, 0, 0) m and (−0.2, 0, 0) m, respectively, and the distance D = 0.4 m. The specific settings are as shown in Figure 3b and the simulation results are shown in Figure 8, Figure 9, Figure 10, Figure 11 and Figure 12. To quantify the reconstruction accuracy of the sound pressure on the reconstruction surface, the percentage of reconstruction error is defined as

(37) $$\mathrm{RE}=\frac{{∥(|{\tilde{p}}_{cal}|-|{\tilde{p}}_{true}|)∥}_{2}}{{∥(|{\tilde{p}}_{true}|)∥}_{2}}\times 100\%,$$

where ${\tilde{p}}_{cal}$ is the calculated sound pressure of the reconstruction surface and ${\tilde{p}}_{true}$ is the theoretical pressure. The lower the reconstruction error is, the higher the reconstruction accuracy is.

### 4.1. Simulation for Single Sound Source

The purpose of this part is to compare the reconstruction performance of LPBCS with the conventional equivalent source method TRESM and the sparse method WBH. The reconstructed sound pressure along the middle row of reconstruction surface and two-dimensional pressure distribution on the reconstruction surface are shown in Figure 4 and Figure 5. Figure 4 mainly compares the accuracy of sound pressure reconstruction, while Figure 5 is mainly used to observe the accuracy of positioning. The frequencies of comparison are 400, 800, 1800, and 3000 Hz with a signal-to-noise ratio (SNR) of 20 dB.

Figure 4 shows that the sound pressure calculated by TRESM at each frequency is much lower than the theoretical sound pressure, which means that the reconstruction accuracy of sound pressure is poor. The sound pressure of WBH has a certain gap with the theoretical sound pressure at low frequencies, and gradually approaches the theoretical sound pressure as the frequency increases. The pressure calculated by LPBCS is similar to the theoretical sound at each frequency, so its reconstruction accuracy of sound pressure is better than other two methods. The purpose of LPBCS-*v* is not to completely reconstruct the sound field but to locate the sound source. LPBCS-*v* can reduce the main lobe width so it can provide a more accurate position than LPBCS.

As Figure 5 shows, the source map of TRESM has a large hot-spot at low frequency, such as 400 and 800 Hz, so the resolution is poor. However, the positioning does not fail. As the frequency increases, the hot-spot becomes smaller but more ghost sources appear, which affect its positioning performance to some extent. The source map of WBH has a slightly larger hot-spot than the result of LPBCS at 400 Hz, so the resolution is slightly worse than LPBCS. Its positioning effect is close to the LPBCS at 800, 1800, and 3000 Hz. These show that WBH and LPBCS have similar effects, and both of them are better than TRESM on reconstruction accuracy and positioning performance. When LPBCS-*v* is used, the main lobe width is reduced while the sound pressure peak is guaranteed. The resolution is greatly improved and the positioning is more accurate.

Figure 6 shows the reconstruction error curves in the frequency range of 400–4000 Hz under different measurement noise levels (i.e., 10, 20, and 30 dB). The reconstruction error for each frequency is calculated 20 times to obtain an average reconstruction error. The results show that the TRESM reconstruction error is large and increases rapidly with the increase of frequency. This is because the number of observations in the high frequency is seriously insufficient and therefore sparse promotion method is needed to enhance the reconstruction accuracy. Obviously, under different measurement noise levels, the reconstruction accuracy of WBH and LPBCS in the frequency band is higher than TRESM. While LPBCS is slightly better than the WBH method at low frequencies, the two methods are similar at high frequencies. Figure 7 shows the reconstruction error curves with different measurement distances (i.e., 0.1, 0.2, and 0.3 m). All methods almost maintain their trends of error curves at different measurement distances. Moreover, the results show that LPBCS can maintain a better reconstruction accuracy and maintain its low-frequency accuracy advantage under different measurement distances.

### 4.2. Simulation for Coherent Sound Sources

It is necessary to distinguish the pressure generated by the coherent sound sources. Figure 8 shows the reconstructed pressure of coherent sound sources along the middle row of reconstruction surface. Figure 9 and Figure 10 show the two-dimensional pressure distribution of the coherent sound sources. In order to strengthen the research on the reconstruction accuracy and localization accuracy of coherent sound sources, more frequencies are used for comparison. The frequencies of comparison are 400, 600, 800, 1000, 1200, 1800, 3000, and 4000 Hz.

It can be seen from Figure 8 that the sound pressure calculated by TRESM is significantly different from the theoretical sound pressure at each frequency, and the sound pressure reconstruction results are very poor. For WBH, the reconstructed and theoretical sound pressure are also different from 400 to 1000 Hz. When the frequency is increased to 1200 Hz, the reconstructed sound pressure of WBH is close to the theoretical value, but the amplitude is biased. At 3000 and 4000 Hz, the sound pressure reconstructed by WBH is very close to theoretical sound pressure. Compared with WBH and TRESM, LPBCS has better reconstruction accuracy at the corresponding frequencies. Only LPBCS can reconstruct two main lobes at a low frequency of 400 Hz. At 600 and 800 Hz, the reconstructed sound pressure of LPBCS is close to the theoretical value, although the sound pressure of the sound source is underestimated. It is consistent with the theoretical sound pressure from 1000 to 4000 Hz. For LPBCS-*v*, it cannot reconstruct the true sound pressure but can significantly reduce the width of two main lobes. Hence, it can provide more accurate positions with a high dynamic range.

Figure 9 and Figure 10 present the positioning performance of coherent sources more clearly. TRESM is unable to locate the sound sources at 400, 600, and 800 Hz, and the two sound sources are completely replaced by several larger hot spots. The positioning performance of TRESM is improved when the frequency of the sound source is set at 1000, 1200, and 1800 Hz, but the spacing of the two sound sources is underestimated. The sound sources positions can be located by TRESM at 3000 Hz, although there is a certain gap between the theoretical and reconstructed sound positions. When the frequency of sound sources is set at 4000 Hz, a large number of ghosts seriously affect the positioning accuracy of TRESM. For WBH, the positioning fails at 400, 600, and 800 Hz. To avoid these situations, WBH is recommended to be used above the 0.7 times the frequency of a half wavelength average inter-element spacing. At 1000 Hz, although there are some offsets in the positions of the two sources, the positions of the sound sources can already be identified by WBH. The sources can be accurately positioned by WBH from 1200 to 4000 Hz. For LPBCS, although there is a positioning deviation at the low frequency of 400 Hz, the positioning does not fail. From 800 to 4000 Hz, this method can accurately locate sound source positions, which are consistent with the theoretical sound source positions. By using LPBCS-*v*, the resolution is enhanced, and the positioning performance is significantly improved. It can be seen that the proposed method can significantly improve the positioning accuracy at low frequencies and is more applicable in a wider frequency band.

The reconstruction error curves of the coherent sound sources under different measurement noise levels are shown in Figure 11. TRESM has a large error in the frequency band shown. The sound pressure reconstruction accuracy is poor, and the reconstruction error increases versus frequency. For each different noise level, WBH has a large reconstruction error at low frequencies, but a better reconstruction accuracy at middle and high frequencies. The LPBCS reconstruction error is less than TRESM and WBH throughout the displayed frequency band under different measurement noise levels. Notably, the limitation of lower frequencies accuracy of the LPBCS is smaller than that of conventional WBH. The results show LPBCS reconstructs the sound pressure of the coherent sound sources more accurately than the conventional method, which makes the positioning of the sound sources more accurate. Figure 12 shows the reconstruction error curves with different measurement distances. It can be seen that as the distance changes, the reconstruction error curves of LPBCS can maintain a relatively stable state. The accuracy is better than the traditional ESM and WBH. The advantage of its low-frequency reconstruction accuracy is more obvious. However, although the low-frequency accuracy of LPBCS is better than the WBH method under a large noise level, its error curves fluctuate. There is a certain gap between its low-frequency accuracy and high-frequency accuracy. It can be seen that the improvement of low-frequency accuracy with the large background noise is still an open and challenging issue.

## 5. Experimental Validation

**Figure 13:**
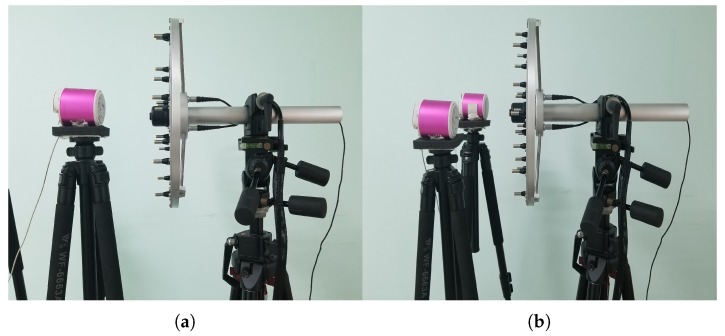
The setup of the experiments for the source reconstruction. (**a**) One loudspeaker; (**b**) Two loudspeakers.

**Figure 14:**
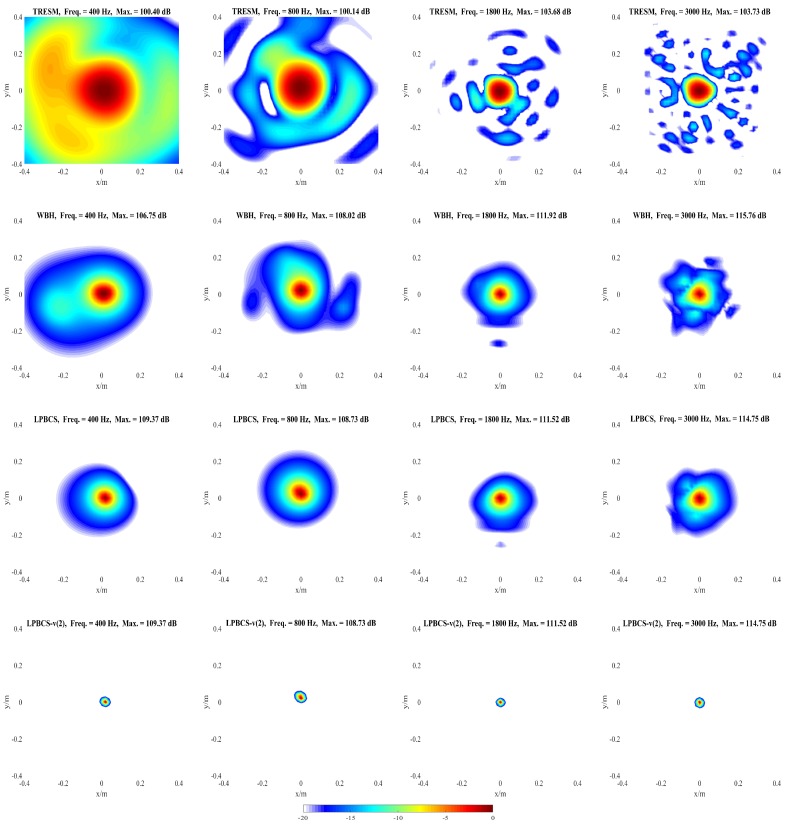
Experimental sound source maps for single source at 400 Hz (**the first column**), 800 Hz (**the second column**), 1800 Hz (**the third column**), 3000 Hz (**the fourth column**). For each column, from top to bottom are TRESM, WBH, LPBCS, and LPBCS-v(2).

**Figure 15:**
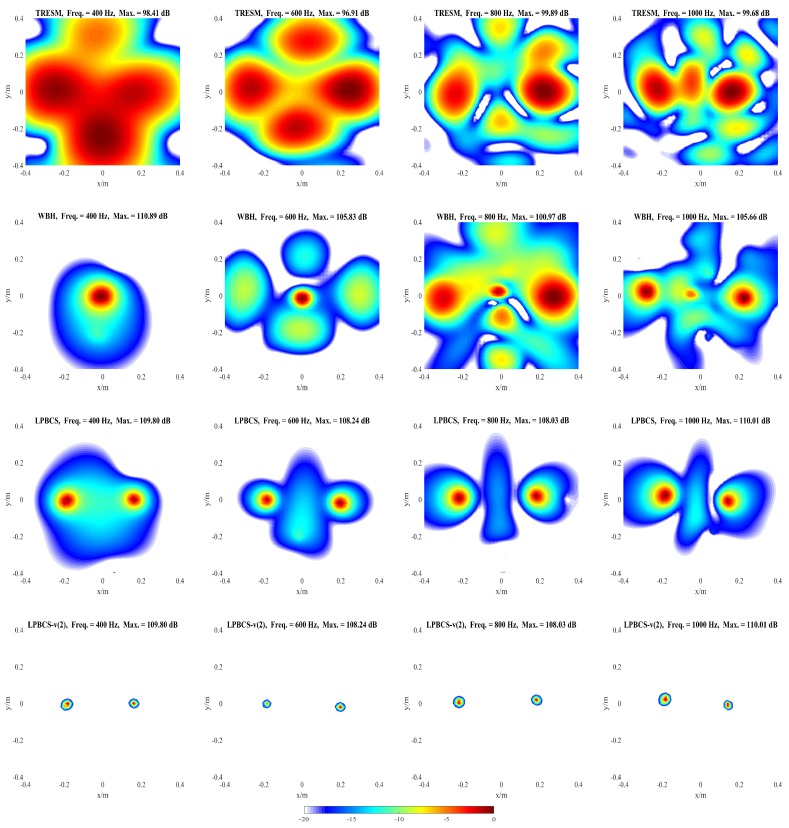
Experimental sound source maps for coherent sources at 400 Hz (**the first column**), 600 Hz (**the second column**), 800 Hz (**the third column**), 1000 Hz (**the fourth column**). For each column, from top to bottom are TRESM, WBH, LPBCS, and LPBCS-v(2).

**Figure 16:**
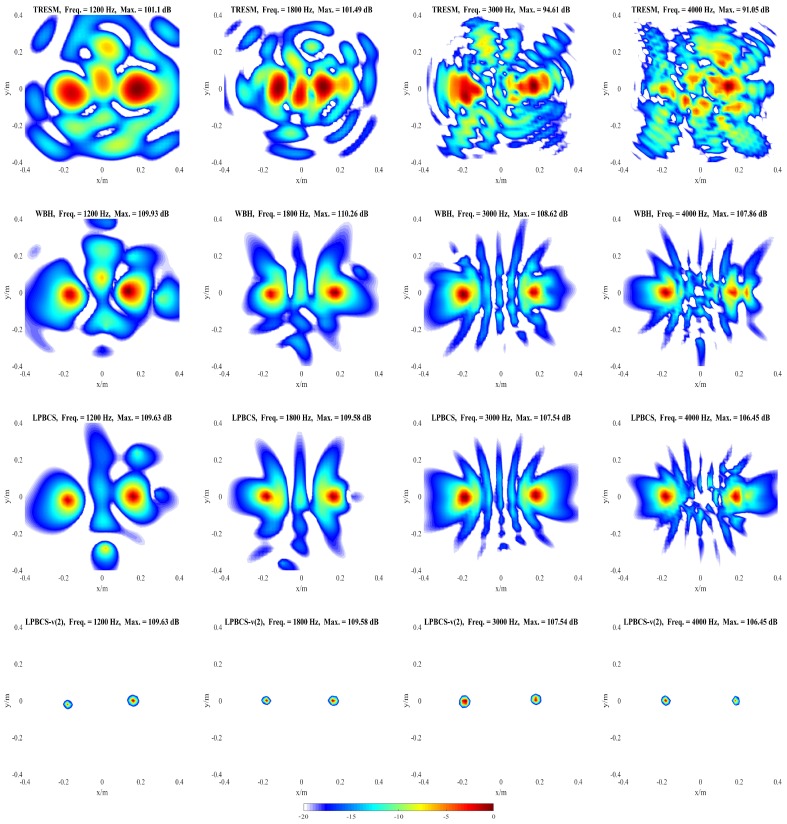
Experimental sound source maps for coherent sources at 1200 Hz (**the first column**), 1800 Hz (**the second column**), 3000 Hz (**the third column**), 4000 Hz (**the fourth column**). For each column, from top to bottom are TRESM, WBH, LPBCS, and LPBCS-v(2).

To assess the applicability of the LPBCS, experimental verification is carried out in this section. Three methods are compared by a couple of experiments, including the single sound source and coherent sound sources. The experimental equipment includes an 18-channel Combo microphone array and a B&K LAN-XI data collector module. Consistent with the simulation, the distance between the speakers and the microphone array is 0.2 m. The locations of the loudspeakers are as shown in Figure 13. When performing a single source test, the loudspeaker is placed in the center of the sound source surface. For coherent sound sources, the two loudspeakers are symmetrically arranged on the sound source plane with an interval of 0.4 m. The in-phase sine waves are excited by loudspeakers. The sampling frequency of the collector is set to 16,384 Hz and the sampling time is 3 s. The meshing and distance settings for the equivalent source and reconstruction surfaces are the same as the simulation. The pressure distribution maps of reconstruction surface are presented in Figure 14, Figure 15 and Figure 16. Their frequencies correspond to the simulation.

As can be seen from Figure 14, for the single source, all methods can identify the location of the loudspeaker. However, at 400 and 800 Hz, the hot-spots identified by the TRESM are larger. At 1800 and 3000 Hz, for TRESM, the range of hot-spots is reduced but the accompanying ghost sources increase. While the maps of WBH have slightly smaller hot-spots at the frequencies shown and provide higher frequency resolution as the frequency increases. Overall, LPBCS maintains a good frequency resolution at the indicated frequencies compared with the other two methods, and its positioning performance is better. The LPBCS-*v* can identify the location more accurately, which is in agreement with the simulation.

Figure 15 and Figure 16 correspond to the recognition result of the coherent sound sources. It can be seen that multiple hot-spots identified by TRESM cannot locate the sound source at low frequencies. A large number of ghosts appear at 3000 and 4000 Hz, which seriously affect the positioning accuracy of TRESM. WBH fails to identify the locations at 400, 600, and 800 Hz. As frequency increases, its positioning performance becomes better. The sources can be accurately positioned by WBH from 1200 to 4000 Hz. It is noteworthy that only LPBCS can locate the coherent sound sources at 400 Hz. Although the peak positions deviate from the actual speaker positions, the deviation is small. LPBCS is able to pinpoint the sound sources at other higher frequencies. Meanwhile, LPBCS-*v* can further improve the frequency resolution and accurately locate the sound sources.

Therefore, similar to the simulation results, LPBCS can effectively identify the coherent sound sources with high accuracy over a wider frequency range. Compared with conventional methods, LPBCS has obvious advantages. However, when the order v>0, if there is a relative energy difference between the two sound sources, the smaller strength source may be covered by the larger strength source. In actual measurements, in order to obtain the maximum dynamic range, the order *v* can be gradually increased to achieve the purpose of accurately identifying the sound source location. In other words, as *v* gradually increases, the range of hot-spots will gradually decrease, which can make the positioning range smaller. So when our goal is to reconstruct the sound field, set *v* to 0, and when we are more interested in the specific location of the sound source, we should increase the order appropriately.

## 6. Conclusions

A sound field reconstruction algorithm LPBCS is suggested to solve the inverse problem based on ESM over a wide frequency range from a Bayesian perspective. The proposed method uses a hierarchical form of Laplace prior on each equivalent sound source and the regularization parameter can change with iterations. The results of numerical simulations show that LPBCS is superior to TRESM in terms of reconstruction accuracy and positioning performance. Compared with WBH, LPBCS has higher precision in coherent sound sources identification. The robustness of the proposed method is verified by changing the frequency, source measurement distance, and noise level. By combining the advantage of function beamforming with LPBCS, LPBCS-*v* can accurately locate the equivalent sources with a high dynamic range. The experimental results are in agreement with the simulation results, which prove the stability and reliability of the proposed method. Valuable and meaningful areas of future research may include the improvement of low-frequency accuracy under large background noise and the development of CS technical for better accuracy.
